# Plasmid-mediated macrolide resistance among rapidly growing mycobacteria in Japan

**DOI:** 10.1093/jacamr/dlag048

**Published:** 2026-04-02

**Authors:** Takeshi Komine, Panuwat Sathianpitayakul, Nobuya Sakagami, Mitsunori Yoshida, Masato Suzuki, Yoshihiko Hoshino, Panan Ratthawongjirakul, Manabu Ato, Hanako Fukano

**Affiliations:** Leprosy Research Center, National Institute of Infectious Diseases, Japan Institute for Health Security, Higashimurayama, Tokyo, Japan; Microbiology Laboratory, Clinical Pathology Department, Ramathibodi Hospital, Bangkok, Thailand; Program of Molecular Sciences in Medical Microbiology and Immunology, Faculty of Allied Health Sciences, Chulalongkorn University, Bangkok, Thailand; Microbiology Testing Department, Special Testing Division, SRL Inc., 50 Fuchigami, Akiruno, Tokyo, Japan; Leprosy Research Center, National Institute of Infectious Diseases, Japan Institute for Health Security, Higashimurayama, Tokyo, Japan; Antimicrobial Resistance Research Center, National Institute of Infectious Diseases, Japan Institute for Health Security, Higashimurayama, Tokyo, Japan; Leprosy Research Center, National Institute of Infectious Diseases, Japan Institute for Health Security, Higashimurayama, Tokyo, Japan; Center of Excellence for Innovative Diagnosis of Antimicrobial Resistance, Faculty of Allied Health Sciences, Chulalongkorn University, Bangkok, Thailand; Leprosy Research Center, National Institute of Infectious Diseases, Japan Institute for Health Security, Higashimurayama, Tokyo, Japan; Leprosy Research Center, National Institute of Infectious Diseases, Japan Institute for Health Security, Higashimurayama, Tokyo, Japan

## Abstract

**Objectives:**

Plasmids carrying the 23S rRNA methylase gene erm(55), which confers inducible macrolide resistance in rapidly growing mycobacteria (RGM), have been reported in the United States. The aim of this study was to investigate the prevalence and genomic characteristics of erm(55)-carrying plasmids in clinically isolated RGM strains in Japan.

**Methods:**

In total, 607 RGM clinical isolates, representing 32 species or complexes, collected between 2019 and 2023 in Japan were examined. Detection of *erm*(55)-carrying plasmids was conducted using PCR screening, minimum inhibitory concentration testing for clarithromycin and whole-plasmid genome sequencing. Comparative genomic analyses were performed to characterize the plasmids.

**Results:**

Five strains belonging to *Mycobacterium murale*, *M. obuense* and *M. chelonae* harboured plasmids carrying the *erm*(55) gene and exhibited inducible macrolide resistance, corresponding to a prevalence of 0.8%. The *erm*(55)-carrying plasmids ranged from 126 187 to 170 220 bp in size. The plasmids showed high overall sequence similarity, and all *erm*(55) sequences were identical to that of the previously reported plasmid pMchErm55 from the United States. Genes predicted to be involved in conjugation-like transfer mechanisms were conserved across all plasmids, whereas regions containing insertion sequence elements and mercury resistance genes exhibited gene content variability.

**Conclusions:**

*erm*(55)-carrying plasmids were identified among clinical RGM isolates in Japan, although their overall prevalence was low. To date, *erm*(55)-carrying plasmids have been identified in at least seven *Mycobacterium* species. Further studies are required to assess their presence among RGM and to clarify their functional characteristics.

## Introduction

The incidence of nontuberculous mycobacterial (NTM) infection is increasing worldwide.^1^ In Japan, the incidence of pulmonary NTM infection was 19.2 per 100 000 people in 2017, exceeding that of tuberculosis.^2^ In Asia, the proportion of rapidly growing mycobacteria (RGM) infections (e.g. *Mycobacterium abscessus* and *Mycobacterium chelonae*) is fairly high among the NTMs.^3,4^

Treatment of NTM infections typically involves multidrug therapy, including macrolide antibiotics such as clarithromycin or azithromycin.^5^ However, several mycobacterial species can exhibit acquired or inducible resistance to macrolides, which is associated with mutations in the 23S rRNA (*rrl*) gene or the 23S rRNA methylase (*erm*) gene, respectively. Acquired resistance-related mutations at positions 2058 and 2059 (*Escherichia coli* numbering) of *rrl* have been reported in *M. abscessus* and *M. chelonae*. The *erm* genes have been identified in various RGMs as follows: *erm*(41) in *M. abscessus*; *erm*(38) in *Mycobacterium smegmatis* and *Mycobacterium goodii*; *erm*(39) in *Mycobacterium fortuitum*, *Mycobacterium boenickei*, *Mycobacterium houstonense*, *Mycobacterium neworleansense* and *Mycobacterium porcinum*; and *erm*(40) in *Mycobacterium mageritense* and *Mycobacterium wolinskyi.*^6^

In 2023, a plasmid (pMchErm55) containing *erm*(55) was discovered in *Mycobacterium chelonae* in the United States. This plasmid has also been found in other RGMs, such as *Mycobacterium iranicum* and *Mycobacterium obuense*.^7^ In this study, we aimed to investigate the prevalence and genomic characteristics of *erm*(55)-carrying plasmids in clinical RGM isolates in Japan.

## Materials and methods

### Bacterial isolates

This study investigated 607 RGM strains isolated from a clinical microbiology laboratory in Japan between 2019 and 2023 (Figure S1, available as Supplementary data at *JAC-AMR* Online, Table S1). The isolates originated from pulmonary (65.6%), extrapulmonary (8.2%) and other specimens (1.2%), whereas the origin of 25.0% of the isolates was unknown. These isolates included 32 different species or complexes that were identified using the MALDI Biotyper system (Bruker, Germany; Supplementary methods). To refine species identification within several complexes, average nucleotide identity analysis was performed.

### PCR screening

RGM isolates were screened by PCR using the primer set *erm*(55)P-F-1 and *erm*(55)P -R-1, targeting the *erm*(55)^P^ gene as described by Brown-Elliott *et al.*^7^ The heat shock protein 65 gene was amplified using the primer sets Tb11 and Tb12^8^ as positive controls (Supplementary methods).

### MIC determination

Inducible phenotypic macrolide resistance was assessed in isolates that tested positive for *erm*(55) by PCR, as well as in the type strain of *M. chelonae* (JCM 6388), using clarithromycin in accordance with the Clinical and Laboratory Standards Institute protocol.^9^ The MIC values were determined on Days 3 and 14.

### Sequencing and plasmid assembly

We extracted genomic DNA from mycobacterial isolates using a previously reported method^10^ and obtained sequencing data using Rapid Barcoding Kit 24 V14 and the P2 Solo platform with the R10.4.1 flow cell (Oxford Nanopore Technologies, UK) (Table S2). Base-calling was performed using Dorado v0.9.0 in super accuracy mode (https://github.com/nanoporetech/dorado/). Reads with >*Q*20 and >5000 bp were then extracted using NanoFilt v2.8.0^11^ and assembled using Flye v2.9.3-b1797.^12^ The plasmid and chromosome genomes were annotated using DFAST (https://dfast.ddbj.nig.ac.jp/) and deposited in DDBJ (Table 1, Table S2). Annotated *rrl* sequences were also examined for point mutations at positions 2058 and 2059 (*E. coli* numbering). The *erm* genes, ranging from *erm*(37) to *erm*(55), were searched against the genomes using BLASTn v2.5.0.^13^

**Table 1.** Results of the screening tests in this study

Strain	s	*erm*(55)^P^ PCR^a^	Clarithromycin MIC (mg/L)		Nucleotide at *rrl* position^b^		*erm*(55)-carrying plasmid assembly^c^	Accession No. for plasmid
			3-day	14-day	2058	2059		
JCM 6388^d^	*Mycobacterium chelonae*	—	0.5	0.5	A	A	NA	NA
SRL2021-127	*Mycobacterium chelonae*	+	2	>16	A	A	+	LC872744
SRL2019-498	*Mycobacterium obuense*	+	2	16	A	A	+	LC872745
SRL2021-291	*Mycobacterium obuense*	+	0.5	>16	A	A	+	LC872746
SRL2023-024	*Mycobacterium obuense*	+	1	>16	A	A	+	LC872747
SRL2023-035	*Mycobacterium murale*	+	8	>16	A	A	+	LC872748

NA, not applicable.

^a^+: The 23S rRNA methylase (*erm*(55)^P^) and heat shock protein 65 (*hsp65*) genes were amplified in the PCR screening tests.

^b^23S rRNA (*rrl*) gene sequence position in *Escherichia coli* numbering.

^c^+: *erm*(55)-carrying plasmid sequence was assembled in long-read sequencing analysis.

^d^Control strain in the MIC test.

### Comparative analysis of erm(55)-carrying plasmids

We performed pairwise BLASTn comparisons among the plasmids assembled in this study and previously reported plasmid pMchErm55 (CP118918.1). The *erm*(55)-carrying plasmids were compared and visualized using Proksee.^14^ In the reference genome, mobile genetic elements and putative horizontally acquired regions were detected using mobileOG-db^15^ and alien_hunter v1.7.^16^

Plasmids were reannotated using Prokka v1.14.6 (https://github.com/tseemann/prokka) and pangenome analysis was performed using Roary v3.13.0 (https://github.com/sanger-pathogens/Roary). Phylogenetic analyses of the six plasmid sequences were then conducted, including construction of a maximum-likelihood tree based on core genes identified by Roary and a recombination-free phylogeny using Gubbins v3.4 (https://github.com/nickjcroucher/gubbins) (Supplementary Methods).

## Results

### Prevalence of the erm(55)-carrying plasmid

In our screening tests, 0.8% (5/607) of the RGM isolates possessed the plasmid containing the *erm*(55) gene and showed inducible macrolide resistance (Figure S1, Table S3). In the PCR screening test for the *erm*(55) gene, five isolates (*M. chelonae* SRL2021-127, *Mycobacterium murale* SRL2023-035 and *M. obuense* SRL2019-498, SRL2021-291 and SRL2023-024) showed positive results (Table 1). In four isolates (*M. chelonae* SRL2021-127 and *M. obuense* SRL2019-498, SRL2021-291 and SRL2023-024), the MIC values changed from susceptible levels (3-day MIC, 0.5–2 mg/L) to resistant levels (14-day MIC, 16–>16 mg/L) (Table 1). *M. murale* SRL2023-035 showed resistance at day 3 (8 mg/L), with increases in MIC values observed at day 14 (>16 mg/L). Five plasmids with the *erm*(55) gene (pErm55Mc1, pErm55Mo1, pErm55Mo2, pErm55Mo3 and pErm55Mm1) were detected from *M. chelonae* SRL2021-127, *M. obuense* SRL2019-498, *M. obuense* SRL2021-291, *M. obuense* SRL2023-024 and *M. murale* SRL2023-035, respectively. No mutations were detected at positions 2058 or 2059 (Table 1, Figure S2).

### Comparison of erm(55)-carrying plasmids

**Figure 1.**
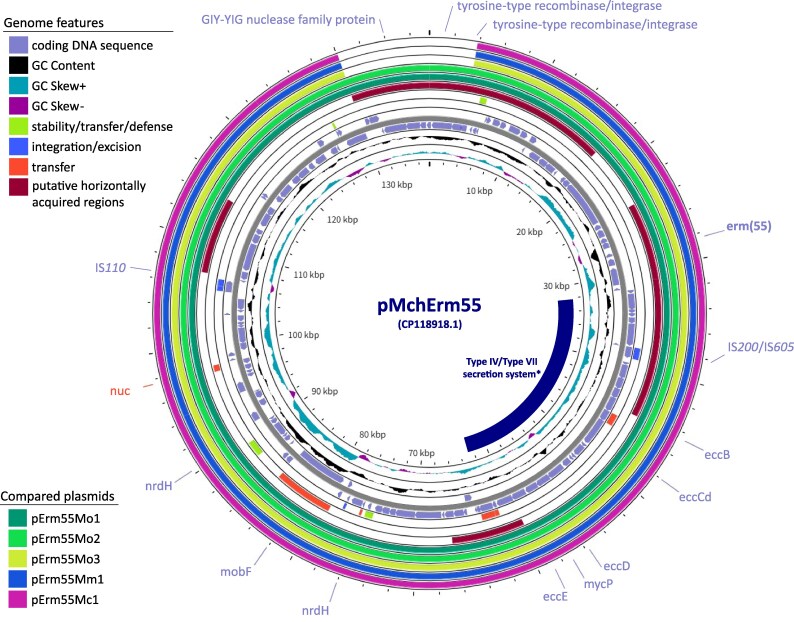
Comparative plasmid maps of *erm*(55)-carrying plasmids, the plasmids used in this study, pErm55Mo1, pErm55Mo2, pErm55Mo3, pErm55Mc1 and pErm55Mm1, and the previously reported plasmid, pMchErm55 (CP118918.1). An asterisk indicates a putative Type IV/Type VII secretion system-associated region, as described in a previous study.^7^ The inner rings show the GC content and GC skew. Highlighted coding DNA sequences, plasmid functionality genes (stability/transfer/defence, integration/excision, transfer) from the mobileOG-db,^16^ and putative horizontally acquired regions detected with alien_hunter v1.7.^17^ Created using ProkSee.com.^15^

The assembled plasmid length was 126 187–170 220 bp, with 119–165 coding DNA sequences (CDSs) (Table S2). Pairwise BLASTn comparisons of the *erm*(55)-carrying plasmids showed weighted percent identity values ranging from 99.5% to 99.9%, with query and subject coverage values ranging from 74.2% to 100% (Table S4). The *erm*(55)^P^ gene sequence (813 bp) was detected in the six plasmids including the reference plasmid, with 100% identity, and was located within a putative horizontally acquired region (Figure 1). All plasmids conserved genes associated with relaxase and type IV and/or type VII secretion systems, including the MOB_F family relaxase (*mobF*) and ESX secretion-associated conserved component (*ecc*) genes, which are implicated in potential conjugation-like transfer mechanisms (Figure 1, Table S5).

While these functional regions were conserved, structural variation was observed in other regions of the plasmids. Plasmids pErm55Mo3, pErm55Mc1 and pErm55Mm1 lacked a region comprising nine CDSs, corresponding to nucleotide positions 1–3848 bp and 130 065–137 526 bp of pMchErm55 (CP118918.1) (Figure 1). This region included CDSs annotated as recombination- and mobility-related proteins. Various insertion sequence (IS) elements were identified across all plasmids (Table S5). The plasmids pErm55Mm1 and pErm55Mo2 harboured mercuric reductase (*merA*) and organomercurial lyase (*merB*), which are involved in mercury resistance.

Pangenome analysis detected 114 core genes among the six plasmids (Figure S3). In the core gene-based and recombination-free phylogeny (Figure S3), pMchErm55, identified from *M. chelonae*, was more closely related to pErm55Mm1 and pErm55Mo plasmids from other mycobacterial species than to pErm55Mc1, which was also derived from *M. chelonae*.

## Discussion

In this study, five isolates from three mycobacterial species, *M. chelonae*, *M. obuense* and *M. murale*, showed inducible phenotypic macrolide resistance associated with *erm*(55)-carrying plasmids. At 0.8% (1/125), the proportion of *erm*(55)-positive isolates among *M. chelonae* clinical isolates in Japan from 2019 to 2023 was lower than that reported in the USA from 2019 to 2021 (3.8%).^8^  *M. murale* can also harbour *erm*(55)-carrying plasmids. Although *M. obuense* and *M. murale* rarely cause human infections,^17,18^ the presence of the *erm* gene warrants consideration in therapeutic decision-making.

Comparative analysis in this study showed that plasmids from different mycobacterial species were often more closely related than those from the same species, supporting the potential for interspecies transmission.^7,19^ Furthermore, the presence of drug and heavy metal resistance genes on these large plasmids suggests a broad role in host environmental adaptability.

Several IS elements were identified within the *erm*(55)-carrying plasmids in this study. A previously reported non-functional *erm*(55) gene truncated by an IS element^19^ further supports the role of IS elements in the diversification of *erm*(55)-carrying plasmids. Furthermore, the presence of multiple recombination-related genes potentially facilitates chromosomal–plasmid recombination, contributing to the stabilization and long-term persistence of antimicrobial resistance determinants.

This study had some limitations. The isolates were obtained from a single testing laboratory and may not reflect nationwide trends in Japan. Moreover, this screening approach detected only *erm*(55)-carrying plasmids, and the number of plasmids available for comparison was limited. Although *rrl* point mutations associated with resistance in other *Mycobacterium* species were not detected in *M. murale*, resistance was observed on Day 3, and the underlying mechanism in this isolate remains unclear, warranting further investigation. Despite these limitations, this study demonstrated the existence of macrolide-resistant RGMs related to the *erm*(55) gene-carrying plasmid in Japan.

Based on the findings of this study and recently published reports,^19^  *erm*(55)-carrying plasmids have now been identified in seven mycobacterial species: *M. chelonae*, *M. bacteremicum*, *M. grossiae*, *M. iranicum*, *M. murale*, *M. neoaurum* and *M. obuense*. Further studies worldwide are warranted to thoroughly assess the presence and characterization of this macrolide resistance-conferring plasmid.

## Supplementary Material

dlag048_Supplementary_Data

## Data Availability

The complete chromosome and plasmid sequences for SRL2021-127, SRL2019-498, SRL2021-291, SRL2023-024 and SRL2023-035 were deposited in the DDBJ/ENA/GenBank databases under accession numbers AP043661 and LC872744, AP043662 and LC872745, AP043663 and LC872746, AP043664 and LC872747 and AP043665 and LC872748, respectively.

## Supplementary data

Figures S1 to S3 and Tables S1 to S5 are available as Supplementary data at *JAC-AMR* Online.
