# Supplementary material for: Plasmid-mediated macrolide resistance among rapidly growing mycobacteria in Japan
Source: JAC Antimicrob Resist. 2026 Apr 2;8(2):dlag048. doi: 10.1093/jacamr/dlag048 (PMC13044508; doi:10.1093/jacamr/dlag048)
Supplement: dlag048_Supplementary_Data [file dlag048_supplementary_data.zip › supplementary_3.2.docx]

**Supplementary methods**

**Identification of nontuberculous mycobacteria using matrix-assisted laser desorption ionization–time-of-flight mass spectrometry (MALDI-TOF MS)**

Mycobacterial colonies grown on Middlebrook 7H11 agar were collected, and protein extraction was performed using a bead-beating procedure with acetonitrile and formic acid, as previously described^1^. Mass spectra were acquired using the MALDI Biotyper system (Bruker, Germany). The resulting spectra were compared with reference spectra in the Bruker Mycobacteria Library (version 6.0) to assign species or species-complex level identifications.

**PCR screening**

PCRs in this study were performed in 20 μL mixture containing 12 μL AmpliTaq Gold 360 with GC-enhancer (Thermo Fisher Scientific, Waltham, MA, United States), 6 μL H_2_O, 0.5 μL of 10 nmol forward primer, 0.5 μL of 10 nmol reverse primer, and 1 μL of bacterial suspension as a template under PCR cycles—an initial denaturation step of 10 min at 95°C for activation, followed by 40 cycles of 95°C for 40 seconds, 60°C for 40 seconds, and 72°C for 60 seconds with a final extension step of 72°C for 10 minutes. PCR products were visualised on an agarose gel via electrophoresis.

**Short-read sequencing**

To identify species within several complexes, whole-genome sequencing analyses were performed. DNA extraction, short-read sequencing, and genome assembly were performed as previously described,^2^ with sequencing conducted using the NovaSeq X Plus platform (Illumina, CA, USA). A taxonomy check of genome sequences was conducted using average nucleotide identity analysis with DFAST_QC v0.5.7.^3^

**Pan-genome and recombination-free phylogenic analyses**

Pangenome analysis was performed using Roary v3.13.0 (https://github.com/sanger-pathogens/Roary). Core gene alignment generated using Roary was trimmed using trimAl v1.4. rev15 with the option '-automated1'.^4^ A maximum-likelihood tree was constructed using the best-fitted nucleotide substitution model (HKY+F) in IQ-TREE v2.3.6,^5^ with 1,000 SH-aLRT and ultrafast bootstrap replicates.

Plasmid sequences were analysed using Snippy v4.6.0 (https://github.com/tseemann/snippy) and Gubbins v3.4.^6^ Subsequently, recombination-free phylogeny was generated in IQ-TREE v2.3.6, with the option '-m GTR+ASC' and a 1,000-replicate, bootstrap approximation. Phylogenies were visualised using an Interactive Tree of Life^7^ (Figure S3).

**Supplementary figures**


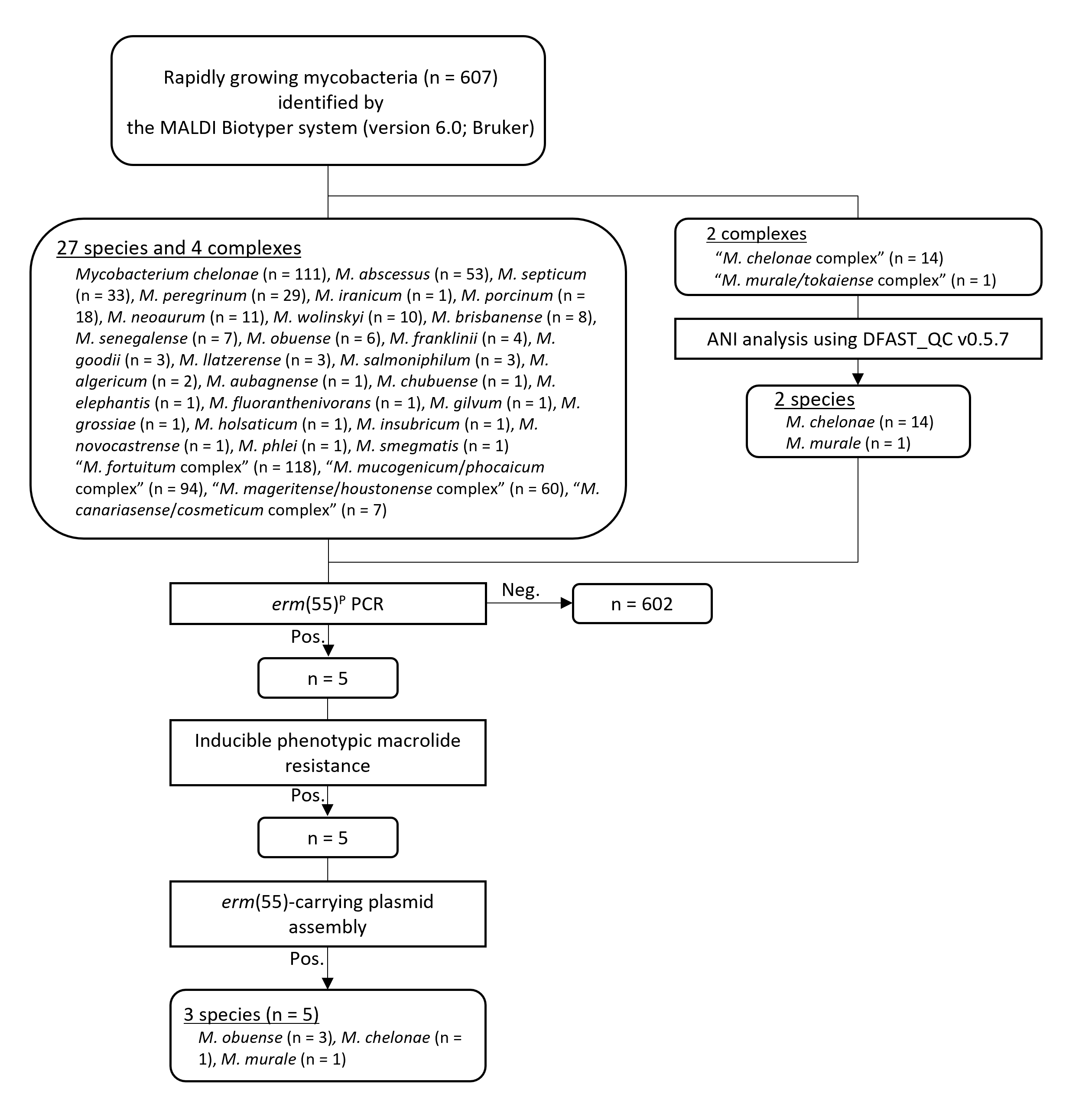


Figure S1. Flowchart of the screening process for the plasmid containing the *erm*(55) gene. ANI, average nucleotide identity.


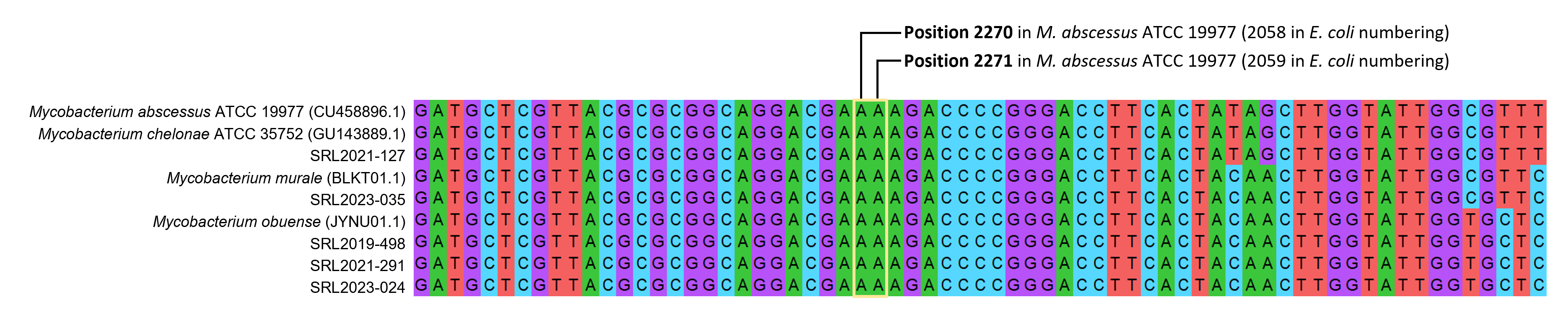


Figure S2. Alignment of 23S rRNA (*rrl*) genes from *erm*(55)-positive isolates and reference *Mycobacterium* strains. Nucleotide positions 2058 and 2059 (*Escherichia coli* numbering), implicated in acquired macrolide resistance, are highlighted in yellow.


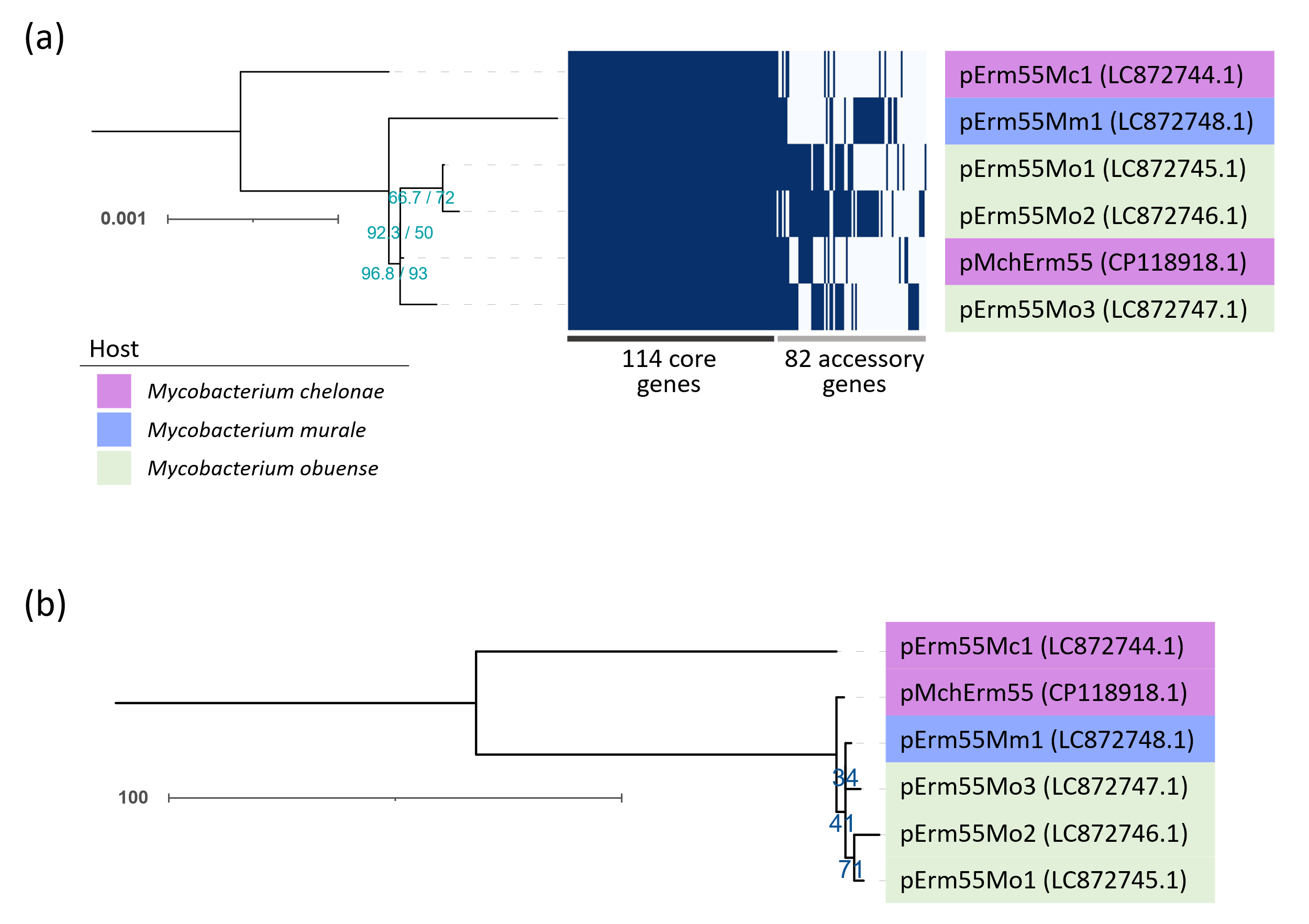


**Figure S3. Genomic comparison of *erm*(55)-carrying plasmids from three mycobacterial species.** (a) Maximum-likelihood tree based on a 114 core gene alignment. The scale bar indicates the number of substitutions per site. Plasmids derived from *Mycobacterium obuense* form a well-supported clade, whereas those derived from *M. chelonae* are distributed across distinct clusters, suggesting limited congruence between plasmid phylogeny and host species. Branch support values are shown as SH-aLRT/ultrafast bootstrap percentages based on 1,000 replicates. (b) Recombination-free phylogeny generated using Gubbins v3.4 shows the same general trend as observed in the core gene–based analysis. The scale bar indicates the number of SNPs. Branch support values are shown as bootstrap percentages based on 1,000 replicates.

**Supplementary references**

1. Toney NC, Zhu W, Jensen B, Gartin J *et al.* Evaluation of MALDI Biotyper Mycobacteria library for identification of nontuberculous Mycobacteria. *J Clin Microbiol* 2022;**60**:e0021722.
2. Komine T, Fukano H, Inohana M *et al.* Draft genome sequences of 25 *Mycobacterium marinum* strains isolated from animals and environmental components in aquaria and an aquaculture farm. *Microbiol Resour Announc* 2022;**11**:e0085122.
3. Elmanzalawi M, Fujisawa T, Mori H *et al.* DFAST_QC: Quality assessment and taxonomic identification tool for prokaryotic genomes. *BMC Bioinformatics* 2025;**26**:3.
4. Capella-Gutiérrez S, Silla-Martínez JM, Gabaldón T. trimAl: A tool for automated alignment trimming in large-scale phylogenetic analyses. *Bioinformatics* 2009;**25**:1972-3.
5. Minh BQ, Schmidt HA, Chernomor O *et al*. IQ-TREE 2: New models and efficient methods for phylogenetic inference in the genomic era. *Mol Biol Evol* 2020;37:1530-4. Erratum in: *Mol Biol Evol* 2020;**37**:2461.
6. Croucher NJ, Page AJ, Connor TR *et al.* Rapid phylogenetic analysis of large samples of recombinant bacterial whole genome sequences using Gubbins. *Nucleic Acids Res* 2015;**43**:e15.
7. Letunic I, Bork P. Interactive Tree of Life (iTOL) v6: Recent updates to the phylogenetic tree display and annotation tool. *Nucleic Acids Res* 2024;**52**:W78-82.
